# College mental health and the digital care transition: national trends in distress, help-seeking, and technology-mediated access, 2020–2025

**DOI:** 10.3389/fdgth.2026.1963340

**Published:** 2026-10-07

**Authors:** Génesis Carolina Arévalo Guadalupe, Luis Alexander Gamboa Tama, Joaquín Estalín Gamboa Tama

**Affiliations:** Universidad Estatal de Milagro (UNEMI), Milagro, Ecuador

**Keywords:** anxiety, college students, depression, digital mental health, health informatics, help-seeking, mental health services, telehealth

## Abstract

**Background:**

College mental health burden increased during the COVID-19 era while university mental health services rapidly adopted remote and digital modes of care. The population-level co-occurrence of these changes has rarely been described across the pandemic and post-pandemic period.

**Methods:**

We performed a secondary ecological trend analysis of seven publicly available, weighted Healthy Minds Study national reports based on successive independent cross-sectional samples from Winter/Spring 2020 through 2024–2025. The reports contained a nominal 553,785 response records across waves; they were not pooled as a single cohort. Harmonized outcomes included depression, anxiety, suicidal ideation, nonsuicidal self-injury, self-reported academic impairment, treatment use, stigma, resource awareness, and selected digital-care indicators. Changes were summarized descriptively as percentage-point and relative differences; no across-wave significance tests or individual-level associations were estimated.

**Results:**

Depression increased from 37% in Winter/Spring 2020 to 44% in 2021–2022 and was 37% in 2024–2025; anxiety increased from 31% to 37% and was 33% in 2024–2025. Self-reported academic impairment of at least 6 days in the prior 4 weeks declined from a Fall 2020 peak of 28% to 16% in 2024–2025. Therapy use increased from 27% to 38%, treatment among students with positive depression/anxiety screens from 51% to 61%, resource awareness from 72% to 83%, and perceived public stigma declined from 51% to 36%. Among therapy users, remote-only care increased from 29% in Fall 2020 to 42% in Winter/Spring 2021, while in-person-only care declined from 35% to 22%; remote-capable care (remote-only plus hybrid) increased from 65% to 77%. In 2024–2025, 7% of the weighted national sample reported a mobile app, online platform, or other digital mental health resource as a provider/resource category.

**Conclusions:**

The observed national estimates show post-peak improvement in several mental health and functional indicators alongside an early-pandemic shift toward remote and hybrid care. Because the analysis used aggregate reports with changing institutional composition, these findings describe population-level trends rather than causal effects or individual recovery.

## 1 Introduction

College student mental health has shifted from a predominantly campus-counseling concern to a system-level public health challenge. Before the coronavirus disease 2019 (COVID-19) pandemic, U.S. surveillance already showed rising mental health diagnoses and service utilization among college students (1, 2). The pandemic then introduced overlapping stressors - social isolation, disrupted routines, bereavement, financial insecurity, health concerns, and abrupt transitions in teaching and care delivery - that were associated with worsening anxiety and depressive symptoms in university populations (3–5). National Healthy Minds Study (HMS) analyses further show that trends differ across racial and ethnic groups, institutions, and sectors of higher education, underscoring that population averages can conceal important disparities in symptom burden and help-seeking (6–9).

The same period also accelerated a second transformation: the digitization of mental health care. Federal and institutional policy changes, together with rapid campus service adaptations, expanded telemental health and hybrid delivery for college students during the COVID-19 pandemic (10–12). For universities, digital mental health is not limited to stand-alone wellness applications. It includes synchronous teletherapy, blended in-person and remote care, web- and app-based self-management, digital navigation tools, online screening, asynchronous support, remote monitoring, and increasingly data-driven approaches. Recent evidence suggests that digital mental health interventions can reduce common psychological symptoms in university students, although effectiveness, uptake, implementation, and maintenance are distinct outcomes (13–16). A 2024 RE-AIM systematic review of 95 college-focused digital mental health intervention studies reported that more than 80% were effective or partially effective for their primary outcome, but uptake was often modest and implementation costs and long-term maintenance were infrequently evaluated (13). A 2025 systematic review and meta-analysis similarly found benefit among university students with existing mental health difficulties while emphasizing heterogeneity in intervention design and study quality (14).

Availability is not equivalent to access or sustained engagement. College students may be willing to consider digital mental health tools yet remain uncertain about privacy, credibility, fit, or the role of an app relative to professional care (13, 17–19). Trials of digital navigation, self-guided app platforms, and multi-component university interventions show that technology can facilitate help-seeking or reduce symptoms, but engagement, context, and implementation strategy materially influence outcomes (20–24). Across digital mental health more broadly, engagement is shaped by usability, perceived usefulness, personalization, privacy, human support, symptom severity, and competing demands (18, 25, 26). Equity also requires deliberate design, including culturally responsive implementation and attention to language, disability, connectivity, device access, and trust (19, 27).

From a clinical and health-systems perspective, the central question is therefore not only whether care became digital, but how the digital transition coincided with changing mental health burden and help-seeking at population scale. Telehealth can extend geographic reach and continuity. At the same time, implementation research and college counseling experience identify challenges involving technology, privacy, workflow integration, crisis response, and follow-up (10–12, 28–31). Digital interventions are therefore best evaluated as components of care pathways rather than as isolated products. Contemporary digital mental health frameworks emphasize integration of telemedicine, online therapy, mobile tools, and human care with safeguards for privacy, security, equity, and clinical accountability (31). University-focused work similarly highlights digital navigation and personalization as emerging components of student mental health services (32, 33). AI-enabled measurement is an additional research direction, but it requires validation and should not be conflated with routine clinical readiness (34–36).

The HMS is uniquely positioned to describe the population context in which this digital-care transition occurred. Its public national reports provide repeated weighted snapshots of depression, anxiety, suicidality, nonsuicidal self-injury, academic functioning, treatment use, stigma, and resource awareness. Importantly for digital health, the reports also captured therapy delivery modality during the early pandemic and, more recently, use of mobile apps, online platforms, or other digital mental health resources. These indicators have rarely been integrated into one longitudinal narrative spanning the acute pandemic and post-pandemic period.

The objective of this study was to harmonize seven public HMS national reports from 2020 through 2025 and characterize parallel changes in (i) mental health symptoms and self-harm indicators, (ii) academic impairment, (iii) treatment use and residual treatment gaps, (iv) stigma and resource awareness, and (v) technology-mediated access to mental health support. The digital-health objective was deliberately narrower than an intervention-effectiveness analysis: we sought to quantify the observed population-level shift in counseling modality from Fall 2020 to Winter/Spring 2021 and to contextualize the 2024–2025 prevalence of reported mobile/online digital mental health resource use. Because only aggregate national estimates were available, no individual-level associations or causal effects were estimated.

## 2 Materials and methods

### 2.1 Study design and data sources

We conducted a secondary ecological trend analysis of seven successive independent cross-sectional HMS national student reports: Winter/Spring 2020, Fall 2020, Winter/Spring 2021, 2021–2022, 2022–2023, 2023–2024, and 2024–2025 (37–43). The reports summarize survey waves conducted at participating U.S. colleges and universities and are publicly available aggregate products of the Healthy Minds Network. Across these reports, the nominal sum of response records was 553,785. This number describes the scale of the source reports only; it is not a pooled cohort size, does not represent 553,785 unique individuals, and was not used as a single analytic denominator.

HMS is a web-based survey of currently enrolled students aged 18 years or older at participating U.S. higher-education institutions. Participating institutions provide a randomly selected sample or, for many smaller institutions, the full eligible student population. Students are invited by email and nonresponders receive reminders. The reports describe nonresponse weighting and equal aggregate weighting of participating institutions so that very large campuses do not dominate national estimates (37–43). Weighting variables, participating institutions, and sampling frames changed across waves. This variation was treated as an intrinsic limitation of serial national surveillance rather than as a source of heterogeneity that could be statistically removed from the public aggregate data.

The source samples ranged from 32,754 respondents in Fall 2020–104,729 in 2023–2024. Response rates across the seven reports were 13%, 14%, 15%, 12%, 9%, 9%, and 8%, respectively (37–43). Thus, the larger respondent counts in several later waves should not be interpreted as improved participation. They reflect changes in the number and size of participating institutions and in institutional sampling frames. For example, the 2023–2024 report included 104,729 respondents from 196 colleges and universities, whereas the 2024–2025 report included 84,735 respondents from 135 institutions (42, 43). The public-report sampling description also changed the threshold at which smaller institutions generally supplied their full eligible population: fewer than 4,000 students in 2022–2023, fewer than 8,000 in 2023–2024, and 12,000 or fewer in 2024–2025 (41–43). HMS applies nonresponse weights to mitigate observable response imbalance, but residual nonresponse bias remains possible.

### 2.2 Mental health and academic measures

Depressive symptoms were assessed with the Patient Health Questionnaire-9 (PHQ-9); we extracted the percentage with scores of 10 or higher, corresponding to moderate-to-severe symptom burden in the HMS reports (44). Anxiety was assessed with the Generalized Anxiety Disorder-7 (GAD-7); we extracted the percentage with scores of 10 or higher (45). These are validated screening thresholds and were interpreted as symptom screens rather than clinical diagnoses.

Additional risk indicators were past-year suicidal ideation and past-year nonsuicidal self-injury (NSSI). Positive mental health was summarized using the Flourishing Scale where comparable (46). Self-reported academic impairment was derived from the HMS item asking how many days in the previous four weeks emotional or mental difficulties had hurt academic performance. We retained two response categories that were stable across reports: ‘6 or more days’ and ‘none.’ This academic-functioning item is a single descriptive HMS self-report indicator rather than a validated psychometric scale comparable with the PHQ-9 or GAD-7; the source national reports do not present a separate validation study for this item. We therefore interpret it only as self-reported functional impact and not as a clinical or diagnostic measure (37–43).

Eating-disorder prevalence was not included in the longitudinal analysis because the HMS changed from the SCOFF screen to the Screen for Disordered Eating in 2024–2025. Exercise was reviewed but not retained as a primary longitudinal outcome because category structures were not sufficiently stable across the seven public reports.

### 2.3 Help-seeking, stigma, and resource awareness

Help-seeking indicators included past-year therapy or counseling, past-year psychiatric medication use, and any past-year therapy/counseling and/or psychiatric medication among students with a positive depression or anxiety screen. We also extracted perceived public stigma, defined in the reports as agreement that most people would think less of someone who had received mental health treatment, and personal stigma, defined as the respondent personally endorsing that view. Resource awareness was the percentage agreeing that they knew where to go to access mental health resources on campus.

To make population-level access patterns more interpretable, we calculated two transparent complements: (i) the untreated positive-screen gap = 100% minus the percentage receiving therapy/counseling and/or psychiatric medication among students with a positive depression or anxiety screen; and (ii) the campus resource-awareness gap = 100% minus the percentage reporting knowledge of where to access campus resources. These derived percentages are ecological descriptors. They must not be interpreted as individual-level unmet need, because the underlying public reports do not allow linkage of symptom severity, perceived need, contraindications, treatment preference, or service use within individual respondents.

### 2.4 Digital-care indicators

The principal digital-care analysis used the therapy-modality item available in Fall 2020 and Winter/Spring 2021. Among respondents who reported receiving counseling or therapy, the reports classified sessions as in-person only, remote/telehealth only (including digital video conferencing and text/app chat), or both in-person and remote (38, 39). These mutually exclusive categories have also been examined in a separate HMS-based study comparing telehealth, hybrid, and in-person campus mental health care (10). Because the categories were mutually exclusive within each wave, we additionally calculated a descriptive ‘remote-capable care’ proportion as remote-only plus hybrid care. No confidence interval was calculated for this derived sum because the covariance structure required to combine the reported category-specific intervals was unavailable.

The 2021–2022, 2022–2023, and 2023–2024 public national reports did not report the same therapy-modality categories in their published appendices and did not contain a directly comparable digital-resource item (40–42). These waves were therefore coded as not reported/not comparable for the digital-care analysis; no values were interpolated. The 2024–2025 report introduced a different marker, ‘mobile app, online platform, or other digital mental health resource,’ among provider/resource types used for therapy or counseling. The weighted national estimate was 7%, with a reported 95% confidence interval of 7%–7% after rounding (43). Because this item allowed multiple provider/resource selections and differs from the mutually exclusive 2020–2021 therapy-modality categories, it was analyzed separately and was not treated as a continuation of the remote-only therapy series.

### 2.5 Data extraction and harmonization

The public reports were reviewed indicator by indicator. Comparability was determined by the author team using explicit criteria: the same underlying construct, denominator, recall period, threshold or category definition, and response structure had to be sufficiently aligned across waves. Labels and published percentages were then cross-checked against the source reports. Indicators meeting these criteria were retained in the longitudinal table. When definitions changed materially, the indicator was excluded from the longitudinal analysis or analyzed as a separate construct. Ambiguous cases were resolved conservatively, and no missing values were interpolated. Digital-care indicators were therefore retained only for the waves in which the public reports provided directly interpretable measures, without treating non-equivalent measures as a continuous time series.

A reproducible extraction file was created with one row per HMS wave and one column per harmonized indicator. A second supplementary file documents the digital-care variables, reported confidence intervals, denominator notes, and comparability constraints. Generative AI was not used to extract, code, harmonize, calculate, or visualize the source data; those steps were completed from the source reports and reproducible Python/Matplotlib workflows.

### 2.6 Statistical analysis

The primary analysis was descriptive. For each longitudinal indicator, we calculated absolute percentage-point changes between clinically or historically meaningful waves and relative change as 100 × (later estimate - earlier estimate)/earlier estimate. We identified observed peaks and minima but did not perform conventional null-hypothesis tests across the seven national waves. This decision reflects three constraints: only seven aggregate observations were available, the waves were not equally spaced, and participating institutions and weighting procedures changed over time. Accordingly, terms such as increase, decrease, peak, and decline refer to observed descriptive differences and not to statistically significant change.

For the digital transition, Fall 2020 and Winter/Spring 2021 were compared using absolute and relative percentage changes in in-person-only, hybrid, remote-only, and derived remote-capable care. Published 95% confidence intervals were reproduced for the three original modality categories but not used to infer causal effects. We did not calculate ecological correlations between mental health burden and digital-care uptake because such correlations would be unstable with seven or fewer aggregate observations and could be misread as individual-level associations.

All figures were generated reproducibly from the harmonized aggregate table using Python and Matplotlib. The figures use distinct markers as well as color to improve interpretability for readers with color-vision differences and were exported at 600 dpi for submission.

### 2.7 Ethical considerations

The underlying HMS surveys involved human participants and the national reports describe institutional review board oversight and confidentiality protections for original data collection (37–43). The present study used only publicly available, de-identified aggregate statistics. The authors did not recruit participants, access identifiable data, or access individual-level HMS records. Ethics approval and informed-consent procedures for original data collection are therefore those described by the Healthy Minds Network in the source reports.

## 3 Results

### 3.1 Study scale and mental health trajectory

Moderate-to-severe depressive symptoms were 37% in Winter/Spring 2020, reached an observed peak of 44% in 2021–2022, and were 37% in 2024–2025 (Table 1; Figure 1). The peak-to-latest difference was −7 percentage points (−15.9% relative to the peak).

**Table 1:** National HMS source samples, response rates, and harmonized mental health/academic indicators.

Wave	N	Response rate	Depression PHQ-9 >=10	Anxiety GAD-7 >=10	Suicidal ideation	NSSI	Academic impairment*
Winter/Spring 2020	55,553	13%	37%	31%	14%	23%	22%
Fall 2020	32,754	14%	39%	34%	13%	23%	28%
Winter/Spring 2021	103,748	15%	41%	34%	13%	23%	27%
2021–2022	95,860	12%	44%	37%	15%	28%	27%
2022–2023	76,406	9%	41%	36%	14%	29%	22%
2023–2024	104,729	9%	38%	34%	13%	26%	21%
2024–2025	84,735	8%	37%	33%	11%	27%	16%

Percentages are weighted national estimates reported by HMS. Response rates are the report-level participation rates. NSSI = nonsuicidal self-injury. *Academic impairment is the self-reported HMS item for 6 or more days in the prior four weeks; it is not a validated scale equivalent to PHQ-9 or GAD-7. The nominal sum of N across reports is not a pooled cohort or unique-person denominator.

**Figure 1:**
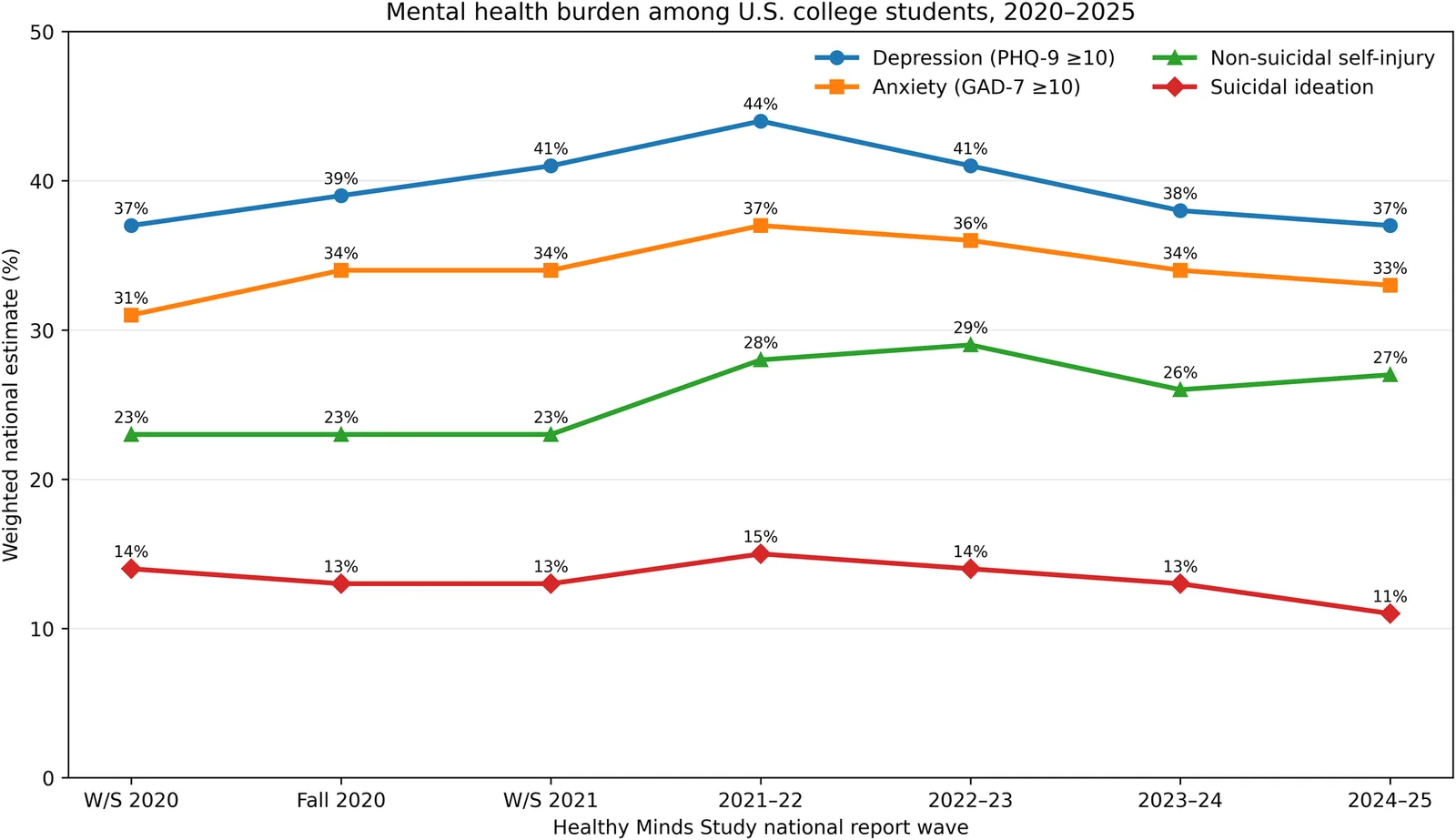
Mental health burden among U.S. college students, 2020-2025. Weighted national estimates of moderate-to-severe depressive symptoms (PHQ-9 >=10), moderate-to-severe anxiety symptoms (GAD-7 >=10), past-year nonsuicidal self-injury, and past-year suicidal ideation across seven Healthy Minds Study national reports.

Moderate-to-severe anxiety symptoms were 31% in Winter/Spring 2020, reached an observed peak of 37% in 2021–2022, and were 33% in 2024–2025 (Figure 1). The peak-to-latest difference was −4 percentage points (−10.8% relative to the peak), while the latest estimate remained 2 percentage points above the earliest 2020 estimate.

Past-year suicidal ideation was 14% in Winter/Spring 2020, reached an observed peak of 15% in 2021–2022, and was 11% in 2024–2025, a −4 percentage-point difference from the peak. NSSI was 23% in Winter/Spring 2020, reached 29% in 2022–2023, and was 27% in 2024–2025, 2 percentage points below the observed peak but 4 points above the earliest estimate. Positive mental health was 39% in Winter/Spring 2020, reached an observed minimum of 32% in 2021–2022, and was 38% in 2024–2025.

### 3.2 Academic functioning and treatment use

Self-reported academic impairment for at least 6 days in the prior four weeks was 22% in Winter/Spring 2020, reached an observed peak of 28% in Fall 2020, and was 16% in 2024–2025 (Table 1; Figure 2). The peak-to-latest difference was −12 percentage points (−42.9% relative to the peak). The ‘no academic impairment’ category was 23% in Winter/Spring 2020, reached a minimum of 17% in Fall 2020 and 2021–2022, and was 33% in 2024–2025.

**Figure 2:**
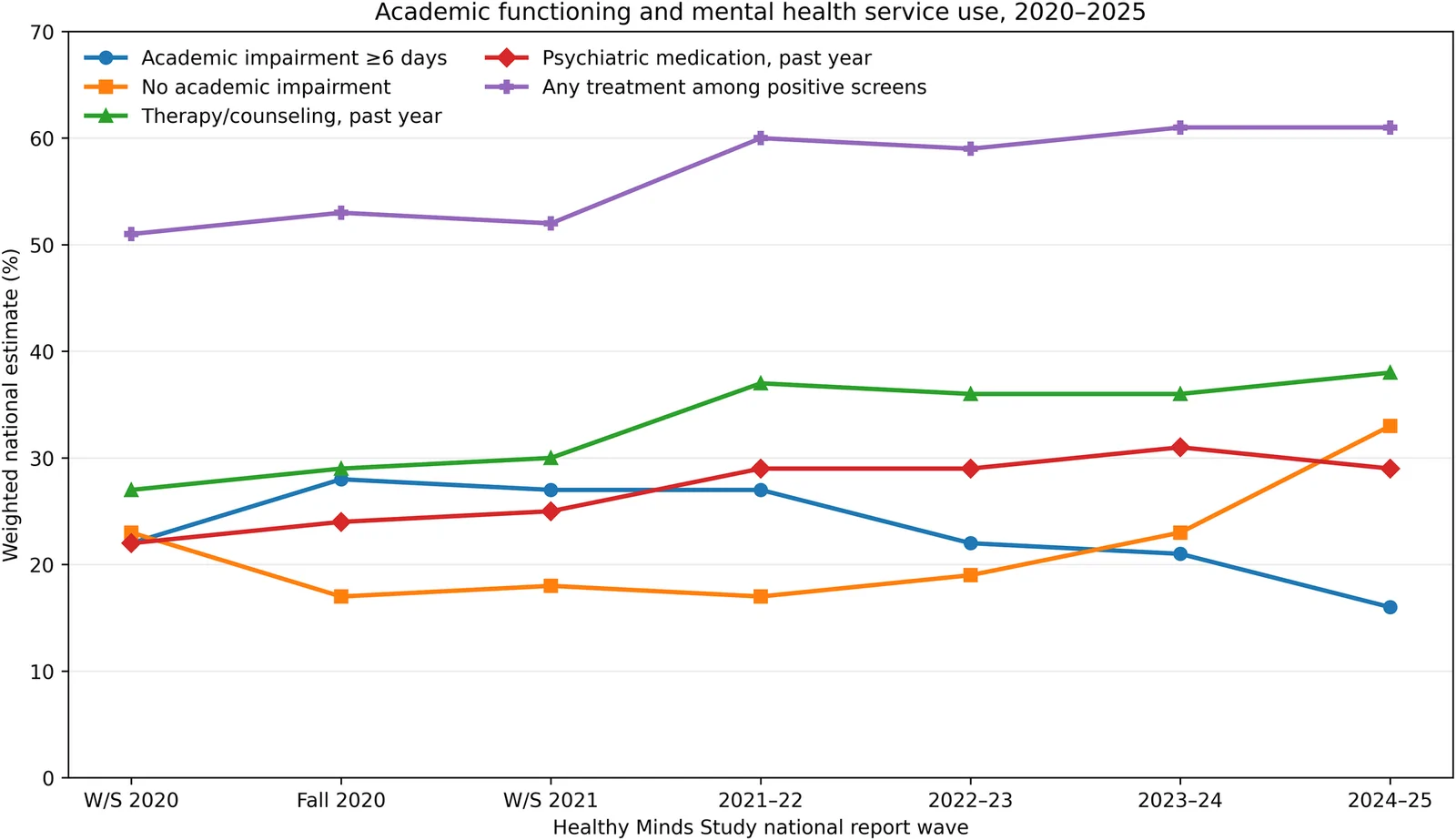
Self-reported academic functioning and mental health service use, 2020-2025. Weighted national estimates of self-reported academic impairment for 6 or more days in the prior four weeks, no academic impairment, past-year therapy/counseling, past-year psychiatric medication, and any therapy/medication among students with a positive depression or anxiety screen. The academic item is a descriptive functional indicator rather than a validated psychometric scale.

Past-year therapy/counseling was 27% in Winter/Spring 2020 and 38% in 2024–2025, a + 11 percentage-point difference (+40.7% relative) (Table 2; Figure 2). Psychiatric medication use was 22% in Winter/Spring 2020, reached 31% in 2023–2024, and was 29% in 2024–2025. Among students with a positive depression or anxiety screen, any past-year therapy and/or medication was 51% in Winter/Spring 2020 and 61% in both 2023–2024 and 2024–2025, a + 10 percentage-point difference from the earliest estimate. The derived untreated positive-screen complement was 49% in Winter/Spring 2020 and 39% in 2024–2025, a −10 percentage-point difference.

**Table 2:** Help-seeking, stigma, resource awareness, and descriptive access gaps.

Wave	Therapy past year	Medication past year	Treatment among positive screens	Untreated-screen gap*	Public stigma	Knows campus resources	Awareness gap*
Winter/Spring 2020	27%	22%	51%	49%	51%	72%	28%
Fall 2020	29%	24%	53%	47%	45%	70%	30%
Winter/Spring 2021	30%	25%	52%	48%	45%	70%	30%
2021–2022	37%	29%	60%	40%	40%	73%	27%
2022–2023	36%	29%	59%	41%	41%	76%	24%
2023–2024	36%	31%	61%	39%	41%	75%	25%
2024–2025	38%	29%	61%	39%	36%	83%	17%

*Derived measures. Untreated-screen gap = 100% minus treatment among positive depression/anxiety screens. Awareness gap = 100% minus knowledge of campus resources. These complements are population-level descriptors and do not establish individual unmet need.

### 3.3 The early-pandemic digital care transition

The strongest direct evidence of a digital-care transition was observed in counseling modality (Table 3; Figure 3). In Fall 2020, 35% of therapy users reported in-person-only sessions (95% CI 34%–37%), 36% reported both in-person and remote sessions (34%–38%), and 29% reported remote/telehealth-only sessions (27%–30%) (38). By Winter/Spring 2021, in-person-only care had fallen to 22% (22%–23%), hybrid care was 35% (34%–36%), and remote-only care had increased to 42% (41%–43%) (39).

**Table 3:** Digital-care markers and non-comparable reporting gaps in the public HMS national reports.

Wave	Digital-care indicator	Estimate	95% CI	Denominator/context	Comparability
Fall 2020	In-person only	35%	34%–37%	Therapy users	Directly comparable with W/S 2021
Fall 2020	Hybrid: in-person + remote	36%	34%–38%	Therapy users	Directly comparable with W/S 2021
Fall 2020	Remote/telehealth only	29%	27%–30%	Therapy users	Directly comparable with W/S 2021
Fall 2020	Remote-capable care*	65%	Not estimated	Therapy users	Derived: hybrid + remote-only
W/S 2021	In-person only	22%	22%–23%	Therapy users	Directly comparable with Fall 2020
W/S 2021	Hybrid: in-person + remote	35%	34%–36%	Therapy users	Directly comparable with Fall 2020
W/S 2021	Remote/telehealth only	42%	41%–43%	Therapy users	Directly comparable with Fall 2020
W/S 2021	Remote-capable care*	77%	Not estimated	Therapy users	Derived: hybrid + remote-only
2021–2022	Directly comparable digital-care marker	NR	NR	Public national report	Not reported/not comparable
2022–2023	Directly comparable digital-care marker	NR	NR	Public national report	Not reported/not comparable
2023–2024	Directly comparable digital-care marker	NR	NR	Public national report	Not reported/not comparable
2024–2025	Mobile app/online platform/other digital MH resource	7%	7%–7%	Weighted national sample	Separate construct; multiple selections allowed

*Derived measure. Remote-capable care is the sum of mutually exclusive hybrid and remote-only categories. No CI was calculated because covariance information was unavailable. NR = not reported in a directly comparable form in the public national report. The 2024–2025 digital-resource item is not directly comparable with the 2020–2021 therapy-modality item; no interpolation was performed.

**Figure 3:**
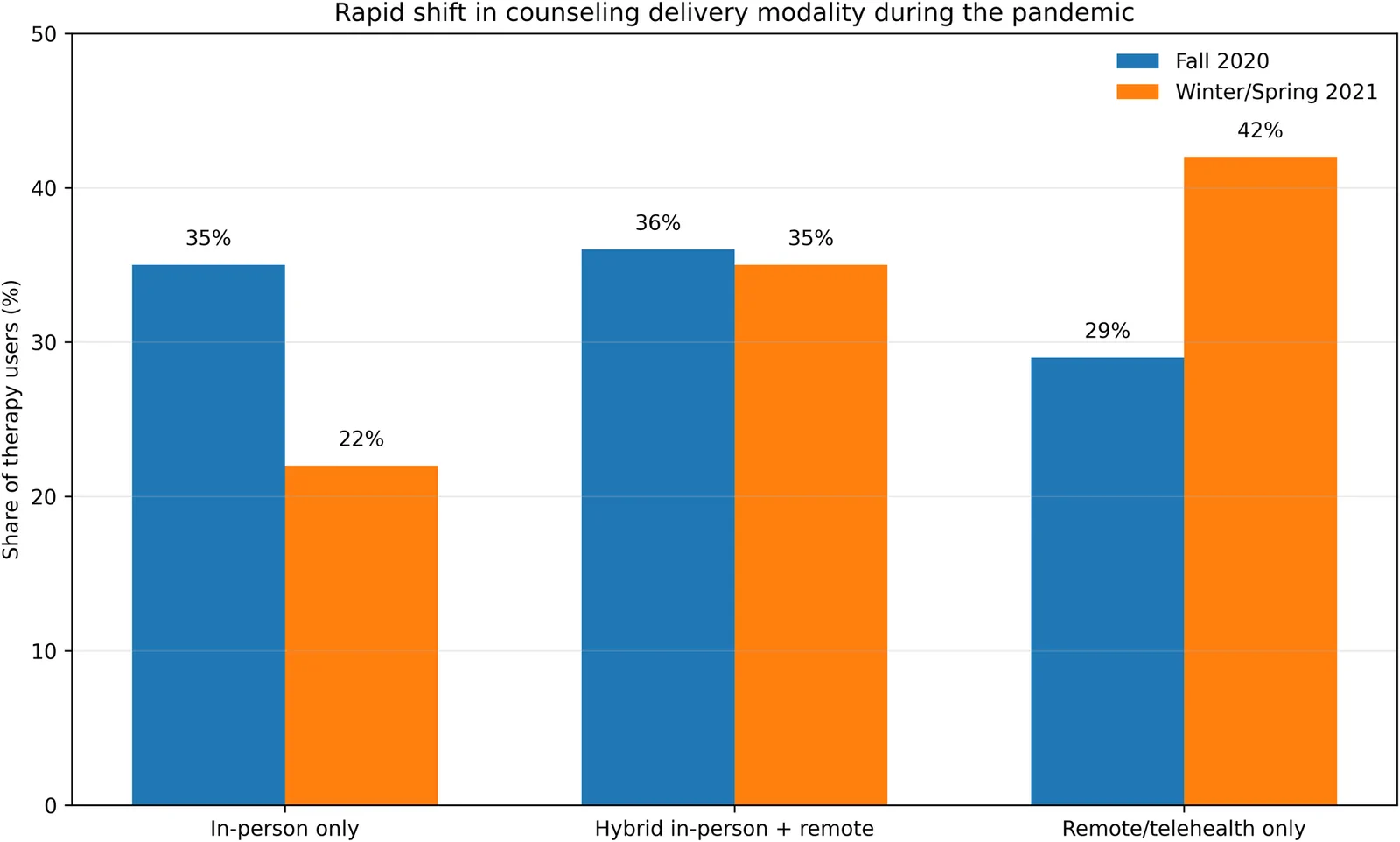
Rapid shift in counseling delivery modality from fall 2020 to winter/spring 2021. Percentages among therapy users reporting in-person-only, hybrid in-person plus remote, or remote/telehealth-only counseling. Values are weighted national estimates reported by HMS; 95% CIs are provided in Table 3.

Remote-only therapy increased from 29% in Fall 2020 to 42% in Winter/Spring 2021, an absolute difference of +13 percentage points (+44.8% relative) (Table 3; Figure 3). In-person-only therapy decreased from 35% to 22% (−13 percentage points; −37.1% relative), while hybrid delivery was 36% and 35%, respectively. The derived remote-capable proportion (hybrid plus remote-only) was 65% in Fall 2020 and 77% in Winter/Spring 2021 (+12 percentage points; +18.5% relative).

### 3.4 Post-pandemic digital resource use and the access environment

In 2024–2025, 7% of the weighted national sample selected a mobile app, online platform, or other digital mental health resource among provider/resource types used for therapy or counseling (95% CI 7%–7% after rounding) (43). In the same report, 38% reported past-year therapy/counseling, 48% reported any past-year medication or therapy, and 61% of students with a positive depression or anxiety screen reported any past-year medication or therapy. The 2021–2022, 2022–2023, and 2023–2024 public national reports did not provide directly comparable digital-care estimates (Table 3).

Perceived public stigma was 51% in Winter/Spring 2020 and 36% in 2024–2025, a −15 percentage-point difference (−29.4% relative) (Table 2; Figure 4). Personal stigma remained between 6% and 7% across all waves. Knowledge of where to access campus mental health resources was 72% in Winter/Spring 2020% and 83% in 2024–2025 (+11 percentage points), while the derived resource-awareness complement decreased from 28% to 17% (−11 percentage points).

**Figure 4:**
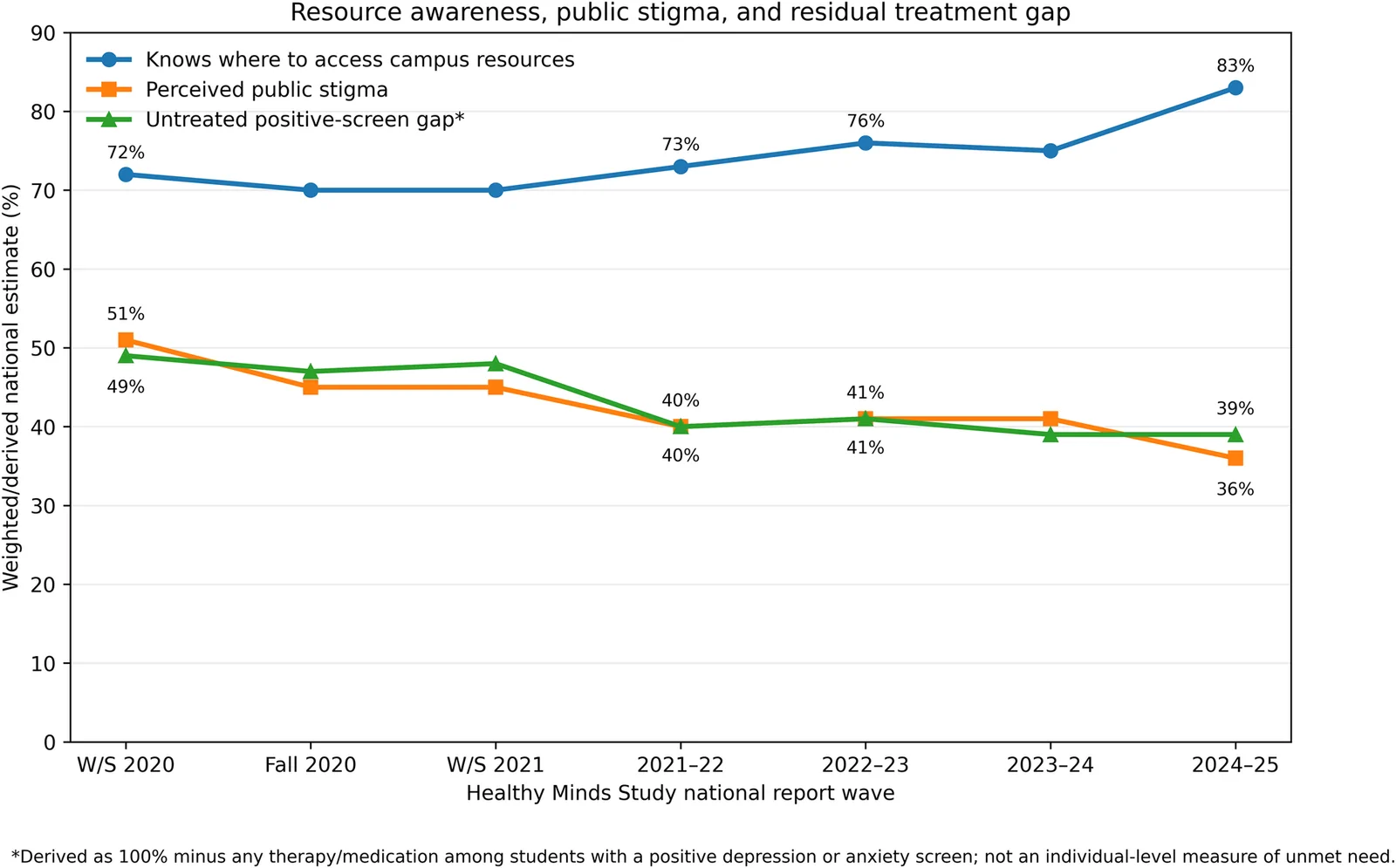
Resource awareness, perceived public stigma, and residual positive-screen treatment gap, 2020-2025. The untreated positive-screen gap is derived as 100% minus the percentage receiving therapy/counseling and/or psychiatric medication among students with a positive depression or anxiety screen and is not an individual-level measure of unmet need.

## 4 Discussion

### 4.1 Interpretation of observed trends and contribution to knowledge

This study describes observed trends across successive independent cross-sectional national samples of U.S. college students; it does not estimate within-person change. Because only aggregate report-level estimates were available and institutional composition changed across waves, conventional significance testing across years was not considered meaningful. Accordingly, the discussion interprets descriptive population-level patterns rather than statistically confirmed temporal effects. Within those constraints, three patterns were evident: several mental health and functional indicators rose to pandemic-era peaks and later declined; treatment use and resource awareness increased while perceived public stigma declined; and counseling delivery shifted sharply toward remote and hybrid modes between Fall 2020 and Winter/Spring 2021.

The main contribution is the integration of these domains within one transparent national trend analysis. Prior work has separately documented changing college mental health burden, increased service utilization, and the rapid adoption of telehealth (1, 2, 6, 10–12). Here, the same public surveillance framework is used to place symptom burden, self-reported academic functioning, help-seeking, stigma, resource awareness, and directly observed counseling modality changes side by side while preserving the limits of non-equivalent digital measures. This provides a population context for digital mental health implementation without claiming that digital care caused the observed changes in symptoms or treatment use.

### 4.2 Clinical interpretation of post-peak changes

The post-peak declines in several indicators are encouraging but should not be equated with clinical recovery or resolution of college mental health need. In 2024–2025, 37% screened positive for moderate-to-severe depressive symptoms, 33% for moderate-to-severe anxiety, 11% reported past-year suicidal ideation, and 27% reported NSSI. Screening instruments do not establish psychiatric diagnoses, yet these values suggest continued need for assessment and mental health services. The wording ‘post-peak decline’ is therefore used instead of ‘recovery’ when describing these aggregate trends.

NSSI showed a different descriptive trajectory from depression, anxiety, and suicidal ideation: the 2024–2025 estimate remained 4 percentage points above the Winter/Spring 2020 value. The aggregate reports cannot determine whether this pattern reflects subgroup composition, disclosure, coping patterns, or different temporal dynamics. It does, however, reinforce the need for future clinical and digital-screening research to assess self-harm directly rather than infer it from depression scores alone.

Self-reported academic functioning provides a complementary but more limited lens. The proportion reporting at least 6 impaired days declined from 28% in Fall 2020 to 16% in 2024–2025, while the proportion reporting no impaired days increased. Because the HMS academic item is a single self-report indicator without the psychometric validation available for PHQ-9 and GAD-7, it should not be interpreted as an equivalent validated outcome. The observed pattern suggests that functional indicators may add context to symptom surveillance, but this proposition requires confirmation with validated academic-functioning measures.

### 4.3 Digital care transition and help-seeking

The early-pandemic modality data document a substantial shift in how therapy was delivered: remote-only care increased from 29% to 42% of therapy users between Fall 2020 and Winter/Spring 2021, while in-person-only care decreased from 35% to 22%. This direction is consistent with contemporaneous evidence describing rapid telehealth adoption in U.S. college mental health services and policy changes that facilitated telemental health during COVID-19 (10–12). The present analysis does not test whether one modality was more effective than another. Instead, it shows that the service channel changed at the same time that mental health burden and help-seeking were changing.

The later 7% mobile/online/digital-resource estimate should be interpreted separately because it is not the same construct as therapy modality. Its value is to show that explicitly digital resources remained one component of the 2024–2025 care ecosystem, not to extend the 2020–2021 telehealth series. Likewise, higher resource awareness and treatment use do not establish that access barriers were resolved. Even in 2024–2025, the descriptive untreated positive-screen complement was 39%; this ecological measure cannot identify individual unmet need, but it highlights the difference between knowing that services exist and actually receiving therapy or medication.

### 4.4 Clinical and service-delivery implications

For clinicians and university health services, the observed trends support evaluating digital care as part of a stepped and coordinated service portfolio rather than as a replacement for face-to-face care. Existing college digital mental health research shows that intervention efficacy does not guarantee uptake, engagement, or sustained implementation (13, 17, 18, 20–26). Teletherapy, guided digital interventions, navigation tools, and self-management resources may address different barriers and levels of need. High-risk presentations still require clear pathways for direct clinical assessment, suicide-risk management, specialty mental health care, and emergency response.

Because the HMS reports did not measure platform architecture or implementation processes, system-design implications should be viewed as author interpretations informed by prior implementation literature rather than as outcomes tested in this study. That literature emphasizes workflow integration, human support, engagement monitoring, privacy, and clinically accountable escalation pathways (11, 12, 18, 28–31). A practical implication for university services is therefore to connect digital screening or navigation to observable referral processes and to measure whether students complete the next step in care, rather than evaluating success only through availability or downloads.

The contrast between high resource awareness (83% in 2024–2025) and the remaining positive-screen treatment complement illustrates why service navigation and clinical capacity should be evaluated together. Possible contributors to non-treatment - including perceived need, wait times, cost, preference, stigma, prior experiences, and treatment readiness - were not jointly measured in the aggregate data and cannot be ranked from this study. Future individual-level or institution-level analyses are needed to determine which mechanisms are most important.

### 4.5 Equity, trust, and digital safety

Digital expansion can reduce some access barriers while reproducing or creating others. College digital mental health studies have often enrolled samples that are disproportionately female and White, limiting evidence about reach across diverse student populations (13). Prior HMS research documents variation across racial and ethnic groups, campuses, and institution types (6–9), while digital mental health research identifies culturally responsive implementation and trust as important determinants of uptake (19, 27). National increases in digital availability should therefore not be assumed to imply equal access or benefit across student groups.

The present study did not measure device access, connectivity, language barriers, disability accessibility, trust in data handling, or subgroup-specific digital engagement. We therefore present these as priorities for future implementation research rather than as explanations of the observed HMS trends. Relevant studies should examine differential onboarding, telehealth attendance, digital engagement, referral completion, and outcomes by demographic and socioeconomic characteristics (19, 27). Privacy and data governance should also be evaluated explicitly when digital tools collect sensitive mental health or behavioral information (28–31).

### 4.6 Future research, including AI-enabled systems

The next analytic step is individual-level HMS microdata or another dataset that can support multivariable and subgroup analyses. Future studies should model year and institution, apply survey weights, assess demographic and socioeconomic effect modification, distinguish synchronous teletherapy from asynchronous digital interventions, and evaluate whether digital engagement is associated with treatment initiation, retention, functioning, or symptom change. Institution-level measures such as wait times, clinician capacity, digital vendor availability, referral completion, and cost would allow implementation and health-services questions that cannot be answered from the public aggregate reports.

AI was not measured as an exposure or intervention in this study and should not be used to explain the 2020–2025 trends. AI-enabled screening, navigation, or decision support is nevertheless a relevant future research area in digital mental health (34–36). Any such work should prospectively test clinical validity, subgroup performance, privacy, safety, and human escalation rather than assuming benefit from technical availability alone.

### 4.7 What this study adds

This study adds a harmonized, reproducible view of seven national HMS reporting periods that links mental health burden, self-reported academic functioning, help-seeking, stigma, resource awareness, and the directly observed early-pandemic shift in counseling modality. Its value lies in showing these domains together while explicitly separating comparable longitudinal indicators from digital measures that changed across reports. The take-home message is therefore descriptive: college mental health indicators improved from several pandemic-era peaks, but substantial symptom burden persisted, while the delivery environment became more treatment-connected and incorporated remote, hybrid, and later explicitly digital resources.

### 4.8 Strengths

Strengths include the large scale of the underlying national reports, coverage of the acute pandemic and post-pandemic period, transparent harmonization rules, explicit reporting of response rates and non-comparable digital waves, reproducible extraction tables, and clear separation between validated symptom screens and less validated functional indicators. The digital-care analysis also uses directly reported counseling-modality categories rather than inferring telehealth exposure from general service use.

### 4.9 Limitations

The limitations define the level of inference. First, the unit of analysis is the national report, not the individual student; no individual-level association, mediation, prediction, causal inference, or within-person change is supported. Second, participating institutions and sample composition changed across waves, introducing potential confounding by campus characteristics such as geography, institutional type, and socioeconomic composition that cannot be controlled with the public aggregate data. Third, response rates were modest and declined in later reports; weighting reduces but does not eliminate nonresponse bias. Fourth, the academic-impairment item is a single self-report indicator and should not be treated as a validated scale equivalent to the PHQ-9 or GAD-7. Fifth, percentages were rounded in the public reports, and the early 2020 and later academic-year reports are not equally spaced observations.

Sixth, digital variables were not reported in a directly comparable form across all waves. The Fall 2020 and Winter/Spring 2021 therapy-modality categories are comparable with each other, whereas the 2021–2022 through 2023–2024 public reports did not provide the same modality measure and the 2024–2025 mobile/online resource item used a different response structure and denominator. We therefore did not interpolate missing digital values or create a continuous digital-use trend from non-equivalent constructs. Seventh, the reports do not identify digital intervention brand, therapeutic content, treatment fidelity, digital literacy, privacy experience, engagement duration, or clinical outcomes attributable to digital care. Eighth, subgroup analyses were not possible from the harmonized aggregate table, so national averages may conceal inequities. Finally, the absence of meaningful across-wave significance testing means that all temporal language should be interpreted as observed descriptive trends rather than statistically confirmed change.

These limitations do not negate the descriptive value of the national series, but they make cautious language essential. The findings are best used to generate implementation and health-services hypotheses that can be tested with individual-level, institution-linked, or prospectively collected data.

## 5 Conclusion

Across seven successive independent cross-sectional HMS national reports from 2020 through 2025, several observed mental health and functional indicators declined from pandemic-era peaks, while substantial symptom burden persisted. During the same period, treatment use and resource awareness increased and perceived public stigma declined. Among therapy users, the directly comparable Fall 2020 and Winter/Spring 2021 data show a marked shift from in-person-only toward remote and hybrid counseling. The 2024–2025 report also documented use of explicitly digital mental health resources, although that item is not directly comparable with the earlier modality measure.

These findings describe population-level trends and should not be interpreted as evidence that digital care caused changes in mental health or treatment use. For university mental health systems, the results support further evaluation of how digital and face-to-face services can be integrated safely, equitably, and measurably. Future work should determine which students engage with digital pathways, who completes referrals, who benefits, and whether technology-mediated care improves outcomes without widening disparities.

## Data Availability

The original contributions presented in the study are included in the article/Supplementary Material, further inquiries can be directed to the corresponding author.
